# Early experience of a dedicated heart–brain integrated ward at Peking Union Medical College Hospital: a retrospective descriptive study

**DOI:** 10.1186/s41016-026-00451-9

**Published:** 2026-10-12

**Authors:** Qi Liu, Yang Zhao, Yanxue Zhao, Sheng Yang, Jun Zheng, Yuanli Zhao

**Affiliations:** 1 Department of Neurosurgery, Peking Union Medical College Hospital, Chinese Academy of Medical Sciences and Peking Union Medical College, Beijing, China; 2 Department of Cardiac Surgery, Peking Union Medical College Hospital, Chinese Academy of Medical Sciences and Peking Union Medical College, Beijing, China

**Keywords:** Heart–brain comorbidity, Integrated care, Multidisciplinary team, Hybrid operating room, Pan-vascular disease, Stroke, Coronary artery disease

## Abstract

**Background:**

Heart–brain comorbidities present unique diagnostic and therapeutic challenges because neurological and cardiac conditions often share pathophysiological mechanisms yet create conflicting treatment demands. Multidisciplinary wards integrating neurosurgery, cardiac surgery, cardiology, and neurology have been proposed, but real-world experience remains limited. We report the initial experience of a dedicated heart–brain integrated ward at a Chinese tertiary referral center.

**Methods:**

We conducted a retrospective descriptive study of all consecutive patients admitted to the Heart–Brain Integrated Ward at Peking Union Medical College Hospital between July 1 and August 21, 2026. Clinical data were entered prospectively into the electronic medical record during routine care and analyzed retrospectively. Variables included demographics, neurological and cardiac diagnoses, a six-category comorbidity classification, MDT decisions, treatment strategies, and discharge outcomes. Treatment categories were predefined as: (i) diagnostic procedures, (ii) therapeutic single-system interventions, (iii) staged dual-system treatment within the same admission, and (iv) synchronous dual-system treatment in a single session.

**Results:**

Fifty-six patients (42 males, 75.0%; mean age 62.4 ± 12.2 years, range 19–80) were enrolled. The most common category was I.1 (shared atherosclerotic vascular disease; 29/56, 51.8%), predominantly ischemic cerebrovascular disease with coronary artery disease (18/56, 32.1%). Category IV (psychocardiological disorders) accounted for 12 patients (21.4%), and Category V (therapeutically interactive) for 3 (5.4%). All patients underwent at least one MDT discussion (100%), and 55 (98.2%) required a second. Of 56 patients, 41 (73.2%) underwent surgical or endovascular intervention and 15 (26.8%) received medical management or remained under evaluation. Among treated patients, 4 (7.1%) received synchronous dual-system procedures, 13 (23.2%) completed staged procedures within the same admission, and 24 (42.9%) underwent single-system intervention. Fourteen patients (25.0%) required intervention for both organ systems. No in-hospital mortality or major procedure-related complications were observed among 38 discharged patients (67.9%); 18 patients (32.1%) remained hospitalized at data cutoff.

**Conclusions:**

Establishing a dedicated heart–brain integrated ward at a tertiary referral center appears operationally feasible and enables structured MDT-based care for complex neuro-cardiac patients. The early disease spectrum is dominated by atherosclerotic pan-vascular disease. Synchronous dual-system procedures were performed in a small selected subset without observed major early events among discharged patients, but this cannot establish safety or generalizability. Larger prospective studies with controlled comparisons and longer follow-up are needed.

## Background

Cardiovascular and cerebrovascular diseases remain leading causes of death and disability worldwide, and their coexistence in individual patients is increasingly recognized as a distinct clinical entity [1, 2]. The pan-vascular medicine concept emphasizes that atherosclerosis can simultaneously affect coronary, cerebral, and peripheral vascular beds [3]. Beyond atherosclerosis, heart–brain interactions encompass stroke–heart syndrome, structural heart disease causing cerebral embolism, and chronic cardiac failure contributing to cognitive impairment [4–8].

Clinical management is complicated by frequent treatment paradoxes: antithrombotic therapy required for coronary disease or atrial fibrillation may precipitate intracranial hemorrhage, while withholding anticoagulation carries thromboembolic risk in patients with mechanical valves [9]. Surgical decision-making is equally challenging, as the optimal timing and sequence of carotid and coronary revascularization remain debated [10].

International initiatives, including the World Stroke Organization’s BEAT program and the European Society of Cardiology consensus on heart failure–stroke interaction, have called for standardized neuro-cardiac care pathways [1]. However, published experience with dedicated physical heart–brain wards remains limited. Peking Union Medical College Hospital (PUMCH) established a Heart–Brain Integrated Ward in July 2026, supported by a joint outpatient clinic, combined cerebro-cardiac imaging, and a hybrid operating room. We report the clinical characteristics, disease spectrum, treatment patterns, and early outcomes of the first 56 consecutive patients.

## Methods

### Study design and setting

This retrospective study involved de-identified clinical data. In consultation with the Institutional Review Board (IRB) of Peking Union Medical College Hospital, the study was deemed exempt from full ethical review, and the requirement for individual informed consent was waived, because the analysis used pre-existing, de-identified clinical records and posed no more than minimal risk.

### Participants and patient flow

All consecutive patients admitted to the Heart–Brain Integrated Ward during the study period were screened. Admission required both a confirmed or suspected neurological condition and a confirmed or suspected cardiac condition, as determined by the receiving attending physician or through referral from the joint outpatient clinic. Patients admitted solely for single-organ evaluation without evidence of comorbidity in the other system were not admitted to the ward and were outside the study cohort. During the study period, 56 patients were admitted; all met eligibility criteria and were included in the analysis. No patients were excluded after admission.

### Data collection and operational definitions

Variables collected included age, sex, admitting department, neurological and cardiac diagnoses, comorbidities, comorbidity category, MDT discussions, treatment strategy, procedures performed, and discharge status. Neurological diagnoses were confirmed by neurosurgeons or neurologists; cardiac diagnoses by cardiologists or cardiac surgeons. We did not systematically collect length-of-stay or cost data for this early analysis.

For descriptive clarity, the following definitions were applied: (1) Diagnostic procedure—cerebral and/or coronary angiography performed solely for diagnostic confirmation; (2) Therapeutic intervention—embolization, angioplasty, stenting, valve implantation, or open surgery with intent to treat a specific lesion; (3) Synchronous dual-system treatment—diagnostic or therapeutic procedures involving both cerebral and cardiac vascular beds performed in a single anesthesia session; (4) Staged dual-system treatment—intervention for one organ system followed by intervention for the other during the same admission; (5) Single-system treatment—intervention directed at only one organ system. A major procedure-related complication was defined as any in-hospital stroke (new focal deficit lasting > 24 h), myocardial infarction, major bleeding requiring transfusion or reoperation, acute kidney injury requiring dialysis, wound infection, or death.

### Comorbidity classification

Patients were categorized using a six-category framework developed jointly by neurosurgeons, cardiologists, cardiac surgeons, and neurologists at our institution before the ward opened (Table 3), adapted from existing literature on pan-vascular disease, stroke–heart syndrome, and cardiogenic embolism [5–8, 11]. The categories are: (I) common etiologic comorbidities (subdivided into I.1 atherosclerotic, I.2 genetic/metabolic, and I.3 infectious/inflammatory); (II) cardiogenic cerebral disorders; (III) cerebrogenic cardiac disorders; (IV) psychocardiological disorders; (V) therapeutically interactive conditions; and (VI) other special/perioperative conditions. Categories are ordered by etiological directionality rather than by severity or prevalence. Patients were prospectively assigned during MDT discussion; when a patient met criteria for more than one category, the category determining the dominant therapeutic decision was selected by MDT consensus. This framework is presented as a proposed conceptual structure requiring external validation.

### MDT and decision-making

Every patient underwent at least one formal MDT discussion attended by attending physicians from neurosurgery, cardiac surgery, cardiology, and neurology, with additional specialists invited as needed. The responsible attending from the primary admitting department led the discussion and communicated the final plan to the patient; in emergency situations requiring immediate action, the most senior available attending from the time-critical specialty made the initial decision, which was reviewed at the next MDT.

### Statistical analysis

Descriptive statistics were used. Continuous variables are presented as mean ± SD or median (range). Categorical variables are presented as counts and percentages. No formal hypothesis testing was performed.

## Results

### Patient demographics

Fifty-six consecutive patients were included. The cohort was predominantly male (42/56, 75.0%), with a mean age of 62.4 ± 12.2 years (median 66, range 19–80). Thirty-one patients (55.4%) were admitted primarily through neurosurgery and 22 (39.3%) through cardiac surgery. Vascular risk factors included hypertension (41/56, 73.2%), hyperlipidemia (18/56, 32.1%), and diabetes mellitus (14/56, 25.0%).

### Disease spectrum

The distribution of comorbidity categories is shown in Table 1. Category I.1 (shared atherosclerotic vascular disease) was the largest group (29/56, 51.8%), predominantly ischemic cerebrovascular disease with coronary artery disease (18/56, 32.1%). Category I.3 (systemic infection/inflammatory etiologies) included 5 patients (8.9%). Category II (cardiogenic cerebral disorders) included 6 patients (10.7%). Category IV (psychocardiological disorders) accounted for 12 patients (21.4%), most commonly patients with comorbid anxiety or stress-related cardiac symptoms in the setting of significant cerebrovascular disease; standardized psychological assessment was not performed and this category requires further clarification in subsequent cohorts. Category V (therapeutically interactive) included 3 patients (5.4%), and Category VI (other special/perioperative) included 1 (1.8%). Among neurological diagnoses, intracranial aneurysm was most frequent (15/56, 26.8%), followed by carotid artery stenosis (12/56, 21.4%), cerebral infarction (11/56, 19.6%), and vertebral artery stenosis (10/56, 17.9%). Among cardiac diagnoses, coronary artery disease was most common (15/56, 26.8%), followed by aortic valve disease (5/56, 8.9%), atrial fibrillation (4/56, 7.1%), and aortic arch pathology (3/56, 5.4%).

**Table 1:** Distribution of heart–brain comorbidity categories

Category	Subcategory	n	%
I. Common etiologic	I.1 Shared atherosclerotic vascular disease	29	51.8
	I.2 Shared genetic/metabolic etiologies	0	0
	I.3 Systemic infection/inflammatory etiologies	5	8.9
II. Cardiogenic cerebral disorders	Cardiac structural/functional disorders causing cerebral injury	6	10.7
III. Cerebrogenic cardiac disorders	Acute/chronic neurological disease with secondary cardiac damage	0	0
IV. Psychocardiological disorders	Psychosocial factors and cardiovascular disease (bidirectional)	12	21.4
V. Therapeutically interactive	Hemorrhagic cerebrovascular disease with cardiac comorbidity	3	5.4
VI. Other special/perioperative	Special states, malignancy, perioperative interaction	1	1.8
Total		56	100

### MDT discussions

All 56 patients (100%) underwent at least one MDT discussion, and 55 (98.2%) required a second. The most frequently debated questions were: (1) whether antithrombotic therapy could be safely continued in patients with intracranial vascular pathology requiring coronary stenting; (2) the sequence of carotid and coronary revascularization; and (3) timing of cardiac surgery in patients with infective endocarditis and cerebral microbleeds.

### Treatment strategies

Of 56 patients, 41 (73.2%) underwent surgical or endovascular intervention, while 15 (26.8%) received medical management or remained under evaluation. Among the 41 treated patients: (1) 4 (7.1% of total cohort) received synchronous dual-system procedures; (2) 13 (23.2%) completed staged procedures within the same admission; and (3) 24 (42.9%) underwent single-system intervention. Fourteen patients (25.0%) required intervention for both organ systems; of these, 4 underwent synchronous procedures and 10 completed staged procedures during the same admission. Thirty-three patients (58.9%) required intervention or medical management for only one system, and 9 (16.1%) were managed medically without planned intervention.

Synchronous dual-system procedures (*n* = 4) included combined cerebral–coronary diagnostic angiography (2), carotid stenting with aortic debranching plus TEVAR in a single session (1), and coronary dilation with intracranial aneurysm embolization (1). Staged procedures within the same admission (*n* = 13) began predominantly with cerebral angiography (8) or coronary angiography (7), followed by second-stage treatment including CABG (4), TAVI (2), aneurysm embolization or clipping (2), and other definitive procedures (5). Single-system procedures (*n* = 24) included aneurysm embolization (5), CABG (2), aneurysm clipping (2), carotid artery stenting (2), and other single-organ interventions (13).

### Discharge outcomes

By the data cutoff (August 21, 2026), 38 patients (67.9%) had been discharged and 18 (32.1%) remained hospitalized. Among the 38 discharged patients, no in-hospital mortality and no major procedure-related complications (as defined above) were observed. Among the 18 patients still hospitalized, no deaths had occurred as of the cutoff; complication surveillance is ongoing (Tables 2 and 3).

**Table 2:** Treatment strategies

Treatment category	n	%
Synchronous dual-system procedure	4	7.1
Staged within same admission	13	23.2
Single-system intervention	24	42.9
Total surgical/endovascular intervention	41	73.2
Medical management/under evaluation	15	26.8

**Table 3:** Proposed six-category classification framework

Major category	Subcategory	Core mechanism	Representative conditions
I. Common etiologic	I.1 Atherosclerotic	Systemic atherosclerosis	CAD + cerebral infarction; multi-vessel bed disease
	I.2 Genetic/metabolic	Gene/enzyme/protein abnormality	Amyloidosis; Fabry disease; CADASIL; HHT
	I.3 Infection/inflammatory	Direct infection or immune inflammation	IE; Takayasu arteritis; vasculitis; sarcoidosis
II. Cardiogenic cerebral	Cardiac → cerebral injury	Embolism, shunt, hypoperfusion	IE + mycotic aneurysm; PFO + paradoxical embolism; AF + ischemia
III. Cerebrogenic cardiac	Brain → cardiac injury	Sympathetic storm, autonomic dysfunction	Stroke–heart syndrome; Takotsubo; epilepsy
IV. Psychocardiological	Bidirectional psych–CV interaction	Autonomic/neuroendocrine activation	Cardiac disease → anxiety; stress cardiomyopathy
V. Therapeutically interactive	Treatment paradox	Anticoagulation vs. bleeding risk	Intracranial aneurysm + CAD/AF; hemorrhagic stroke + anticoagulation
VI. Other special/perioperative	Special states	Aortic, peripartum, perioperative	Aortic dissection + ischemia; Trousseau syndrome

## Discussion

This study describes the first two months of a dedicated heart–brain integrated ward at a Chinese tertiary referral center. The principal findings are: (1) the ward admitted a complex comorbid population with near-universal MDT requirement; (2) atherosclerotic pan-vascular disease dominated the spectrum; (3) dual-system cases were managed predominantly through staged approaches, with a small number of synchronous procedures; and (4) no in-hospital mortality or major complications were observed among discharged patients, though this must be interpreted given incomplete follow-up.

### Comparison with conventional care models

Three care models for heart–brain comorbidity can be distinguished: (1) conventional single-discipline admission with inter-departmental consultation; (2) specialty wards with embedded MDT consultation; and (3) a dedicated integrated ward in which neurosurgery, cardiac surgery, cardiology, and neurology co-manage patients. In this early experience, the practical advantages of model 3 included reduced consultation turnaround, same-access to combined imaging, and the ability to perform synchronous procedures in the hybrid OR without inter-departmental transfer. However, without a controlled comparison, we cannot demonstrate improved clinical outcomes. Challenges included coordinating MDT schedules and training nursing staff to monitor both neurological and cardiac parameters.

### Disease spectrum and the proposed classification

The predominance of Category I.1 (51.8%) is consistent with the known epidemiology of pan-vascular disease [3]. Category IV (psychocardiological disorders) accounted for 21.4% of cases in this early cohort, which likely reflects the referral pattern of the ward during its initial months rather than the true prevalence of psychocardiological disorders in heart–brain comorbidity. Standardized psychological assessment tools were not used, and this category will require refinement in subsequent analysis. Category V (therapeutically interactive, 5.4%) posed the greatest MDT challenges, particularly regarding antithrombotic management [9]. We propose the six-category framework as a conceptual structure for MDT discussion and database coding; it has not been externally validated.

### Treatment decisions encountered in practice

The near-universal need for MDT (100% first, 98.2% second) reflects the complexity of this population. The most frequent practical dilemmas were: (1) whether to continue antiplatelet therapy in patients with unruptured intracranial aneurysms requiring coronary stenting; (2) the sequence of carotid and coronary revascularization in patients with symptomatic bilateral stenosis; and (3) timing of valve surgery in patients with infective endocarditis and cerebral microbleeds. Ten of 14 dual-system patients were treated with staged procedures, consistent with the conservative approach in the literature [11–14]. The four synchronous cases—two combined diagnostic angiographies, one one-session carotid stenting + debranching + TEVAR, and one coronary dilation + aneurysm embolization—were selected for technical convenience or because acute lesions in both systems could not be safely staged [15]. No major early events were observed in these four patients among discharged cases, but the sample is too small to draw conclusions about safety or generalizability.

### Organizational lessons

Several operational lessons emerged. First, clear admission criteria are essential: admission was appropriate when both organ systems had active or high-risk conditions requiring coordinated management, or when a comorbidity materially altered the risk–benefit balance of a planned procedure. Patients with only incidental, low-risk comorbidities were better served in conventional specialty wards. Second, nursing staff required orientation to dual-system monitoring. Third, the hybrid OR served as a diagnostic–therapeutic bridge: several patients underwent combined cerebral–coronary angiography in the hybrid suite, allowing immediate MDT adjustment of the treatment plan.

### Limitations

Several limitations must be acknowledged. First, this is a single-center, retrospective descriptive study with a small sample and short follow-up; it does not compare outcomes with conventional care and cannot establish clinical effectiveness. Second, the patient population is selected for complexity and may not represent the broader comorbidity population. Third, outcome data are incomplete: 32.1% of patients remained hospitalized at data cutoff, and the absence of observed complications among discharged patients should not be extrapolated to the full cohort. Fourth, the six-category classification is proposed based on early experience and requires external validation; Category IV in particular needs further clarification with standardized assessment. Fifth, we did not collect systematic length-of-stay or cost data. Finally, no AI or machine-learning tools were used in this analysis.

### Future directions

A Plan–Do–Study–Act (PDSA) cycle could compare operational metrics—consultation response time, MDT scheduling efficiency, procedure cancellation rates, and length of stay—before and after ward implementation. Prospective multicenter registries with longer follow-up are needed to validate the classification and compare synchronous versus staged strategies. Specific populations warrant further study, including elderly patients with chronic subdural hematoma and concurrent atrial fibrillation or coronary stents [16], and patients undergoing mechanical thrombectomy who require simultaneous cardiac evaluation.

## Conclusions

The first two-month experience of a dedicated heart–brain integrated ward at PUMCH demonstrates operational feasibility: the ward admits a complex comorbid population, achieves universal MDT review, and performs staged and selected synchronous dual-system procedures without observed major early events among discharged patients. The disease spectrum is dominated by atherosclerotic pan-vascular disease. These early descriptive findings are hypothesis-generating. Larger prospective, multicenter, controlled studies with longer follow-up are needed to determine whether this care model improves clinical outcomes.

## Data Availability

No datasets were generated or analysed during the current study.
